# Outdoor Adventure and Experiential Education and COVID-19: What Have We Learned?

**DOI:** 10.1177/10538259211050762

**Published:** 2022-09

**Authors:** Aaron M. Leonard, Alan W. Ewert, Kodiak Lieberman-Raridon, Denise Mitten, Erik Rabinowitz, S. Anthony Deringer, Forrest Schwartz, Steve Smith, Christine L. Norton, John Regentin, Sherry Bagley, Ileana Anderson

**Affiliations:** 1 31713 Prescott College; 2 116724 Sierra Club; 3 Indiana University; 4 1801 Appalachian State University; 5 7174 Texas State University; 6 Experiential Consulting, LLC; 7 FCCS Consulting; 8 3507 Gettysburg College; 9 93318 Association for Experiential Education; 10 University of Utah

**Keywords:** outdoor adventure education, experiential education, wilderness therapy, outdoor behavioral healthcare, COVID-19 pandemic

## Abstract

**Background:** The severe acute respiratory syndrome coronavirus 2 (SARS-CoV-2) now known as COVID-19 changed the world and the outdoor adventure and experiential education (OAEE) fields were not immune. These changes significantly impacted various OAEE programs in multiple ways and at different levels of intensity. **Purpose:** The purpose of this study was to ascertain the impacts of the COVID-19 pandemic on the OAEE fields and identify how OAEE organizations have responded to those impacts. **Methodology/Approach:** Using a three-phase study and a multi-method approach to data collection and analyses, respondents from 115 OAEE organizations (N=115) were asked to indicate how and in what ways their organizations have been impacted by the pandemic and in what ways their organizations have responded to those challenges. **Findings/Conclusions:** Many organizations responded not being ready for the impacts from the COVID-19 pandemic. Significant impacts were noted from most of the OAEE organizations responding and included closings, staff reductions, and downsizing as well as operational changes. Many organizations reported ways they are attempting to mitigate the pandemic effects. **Implications:** Important questions were raised in this paper as to how well prepared the OAEE fields might be for the next crisis.

In numerous ways, the coronavirus-2019 (COVID-19) pandemic represents a perfect storm for the outdoor adventure and experiential education (OAEE) fields on three operational dimensions: timing, economic impacts, and enrollment in programs. In January of 2020, the virus was detected in humans, first in China, then elsewhere (World Health Organization, 2021). The appearance of the virus began to resonate with both public awareness and as a small but growing concern in OAEE programs (Association of Outdoor Recreation and Education, 2020). This is also the timeframe in which many OAEE programs see seasonal enrollment, recruit staff, and begin to plan for summer operations in the Northern hemisphere and winter operations in the Southern hemisphere.

By February of 2020, public awareness was in a steep upward trajectory relative to both the presence and threats posed by the virus (U.S. Department of Health & Human Services, 2020). Mitigation techniques such as physical distancing and hand washing became more evident in both the media and in government proclamations, although it was not until April that the Centers for Disease Control and Prevention (CDC) recommended wearing cloth masks while in public (Firth, 2020). At the same time there was increasing concern regarding the economic impacts the virus might have across the globe (Bluedorn et al., 2020).

By March, the accumulating effects of COVID-19 were apparent in a variety of dimensions including the number of cases and world-wide travel and economic impacts. Close-downs and stay at home orders were becoming the norm and began to exert dramatic effects on the OAEE fields, both in terms of reduced numbers of enrollees and canceled or withdrawn registrations. Serious questions began to be asked as to whether OAEE programs could fulfill anticipated program enrollments, with an increasing number of OAEE organizations reported having to close due to stay at home orders and campus closures, not knowing when or if they would reopen (Colorado Outdoor Recreation Industry Office, 2020; Outdoor Recreation Roundtable, 2020).

April and May of 2020 saw cumulative cases in the United States increasing from 256,503 to 1,863,787 and cumulative deaths increasing from 6,440 to 107,907 (Centers for Disease Control and Prevention, 2021). This increase in cases and deaths, combined with mixed and sometimes competing government efforts to reopen parts of the United States economy, caused an increase in confusion and a range of different regional responses (Tankersley, 2020). There was also increased attention concerning safe operations of day and multi-day camps (American Camp Association, 2020) as well as efforts to develop resources, such as the Young Men’s Christian Association (YMCA) and American Camp Association guide for opening summer camps, to help mitigate the impacts of COVID-19 for the OAEE fields (Association of Outdoor Recreation and Education, 2020; Chang, 2020). However, a substantial number of OAEE organizations reduced their offerings, closed for the upcoming season, or shut-down their program permanently (Colorado Outdoor Recreation Industry Office, 2020; Outdoor Recreation Roundtable, 2020).

July 2020 saw a rapid increase in positive test results with a second COVID-19 peak occurring in the United States on July 24. COVID-19 cases appeared to level out through mid-September, increasing hope that there would be a decrease in cases. Early October, however, saw cases increasing, peaking again in early January 2021, with the United States leading the world in COVID-19 cases and deaths (Centers for Disease Control and Prevention, 2021; Leatherby, 2020; Van Beusekom, 2020). On December 31, 2020, the World Health Organization (WHO) approved the first vaccine for emergency use, and by mid-February seven vaccines approved for use with over 200 in development (World Health Organization, 2020, 2021).

In numerous ways, the COVID-19 pandemic represents a perfect storm for the outdoor adventure and experiential education (OAEE) fields on three operational dimensions: timing, economic impacts, and enrollment in programs. In January of 2020, the virus was detected in humans, first in China, then elsewhere (World Health Organization, 2021). The appearance of the virus began to resonate with both public awareness and as a small but growing concern in OAEE programs (Association of Outdoor Recreation and Education, 2020). This is also the timeframe in which many OAEE programs see seasonal enrollment, recruit staff, and begin to plan for summer operations in the Northern hemisphere and winter operations in the Southern hemisphere.

By February of 2020, public awareness was in a steep upward trajectory relative to both the presence and threats posed by the virus (U.S. Department of Health & Human Services, 2020). Mitigation techniques such as physical distancing and hand washing became more evident in both the media and in government proclamations, although it was not until April that the CDC recommended wearing cloth masks while in public (Firth, 2020). At the same time the International Monetary Fund expressed increasing concern about the global economic impacts of the virus (Bluedorn et al., 2020).

By March, communities around the globe began to see the accumulating effects of COVID-19 in a variety of dimensions, including with the number of cases and impacts on world-wide travel and the global economy. Close-downs and stay at home orders were becoming the norm and began to exert dramatic effects on the OAEE fields, both in terms of reduced numbers of enrollees and canceled or withdrawn registrations. OAEE programs began to question whether they could fulfill anticipated program enrollments, with an increasing number of OAEE organizations reported having to close due to stay at home orders and campus closures (Colorado Outdoor Recreation Industry Office, 2020; Outdoor Recreation Roundtable, 2020).

In April and May of 2020 COVID-19 cases in the U.S. increased from 256,503 to 1,863,787 with cumulative deaths increasing from 6,440 to 107,907 (Centers for Disease Control and Prevention, 2021). This increase in cases and deaths, combined with mixed and sometimes competing government efforts to reopen parts of the United States economy, caused an increase in confusion and a range of different regional responses (Tankersley, 2020). OAEE programs were also devoting increasing attention to the safe operations of day and multi-day camps (American Camp Association, 2020) as well as efforts to develop resources, such as the YMCA and American Camp Association guide for opening summer camps, to help mitigate the impacts of COVID-19 for the OAEE fields (Association of Outdoor Recreation and Education, 2020; Chang, 2020). By that point, however, a substantial number of OAEE organizations had reduced their offerings, closed for the upcoming season, or shut-down their program permanently (Colorado Outdoor Recreation Industry Office, 2020; Outdoor Recreation Roundtable, 2020).

By July 2020, the national tally of positive test results increased, peaking for a second time in the United States on July 24. After leveling off in September 2020, by early October cases were on the rise again, peaking in January 2021 with the United States leading the world in COVID-19 cases and deaths (Centers for Disease Controland Prevention, 2021; Leatherby, 2020; Van Beusekom, 2020). The World Health Organization (WHO) approved the first vaccine for emergency use on December 31, 2020, and by mid-February seven vaccines were approved for use with over 200 in development (World Health Organization, 2020, 2021).

## Related Literature

A growing body of literature suggests the COVID-19 pandemic has had significant consequences for many programs including those of OAEE fields (Quay et al., 2020). Although the pandemic's full impact remains unknown at this time, OAEE programs throughout the world have reported significant financial losses due to the pandemic's economic impacts (Collins et al., 2020; Colorado Outdoor Recreation Industry Office, 2020; Outdoor Recreation Roundtable, 2020). With a goal of ascertaining the effect of COVID-19 upon OAEE programs, early research into the immediate responses to COVID-19 conducted by various organizations between February through July highlighted the initial and anticipated challenges faced by the outdoor industry at large.

For example, the Lawrence Hall of Science at the University of California, Berkeley, conducted a study in the United States in April 2020 on the impact of COVID-19 on environmental and outdoor science education programs (Collins et al., 2020). Their respondents indicated that if social distancing guidelines remain in effect through December 31st, 2020, only 22% of the programs reported that they will be able to reopen, and only 15% reported that they would likely reopen. The study results indicate that if restrictions remained in effect through the end of 2020 an estimated 11 million students would miss out on environmental education opportunities and organizations would lose an estimated $600 million in revenue along with having to furlough 30,000 staff members.

A survey conducted in May 2020 by the Outdoor Recreation Roundtable (ORR), in partnership with Oregon State University Outdoor Recreation Economy Initiative, suggested that the impacts of the pandemic significantly worsened since May 2020. ORR represents over 100,000 recreational vehicles, camping, boating, fishing, horseback, hunting, skiing, hiking, and biking businesses nationwide. The May 2020 ORR survey indicated that the outdoor industry is one of the most significantly impacted sectors in the United States with 95% of businesses reporting a significant decrease in revenue and 79% reporting significant financial impacts due to COVID-19. (Outdoor Recreation Roundtable, 2020). Census Bureau reporting from September 2021 indicates that 75% of small businesses supporting the outdoor industry continues to see significant negative impacts due to the COVID-19 pandemic (U.S. Census Bureau, 2021).

In another study conducted by Leave No Trace (LNT) and Pennsylvania State University, Rice et al. (2020) found a mixed situation with respect to participation rates in outdoor recreation activities. Using a sample of activities commonly associated with OAEE including backpacking, camping, climbing, downhill and cross-country skiing, flatwater canoeing, kayaking, or rafting they found levels of participation in a number of these activities declined with the emergence of the pandemic. Rice et al., reported that over 66% of the study respondents anticipated returning to levels and types of participation they previously engaged in after the pandemic subsided (2020). Another finding from their longitudinal study suggested that at the onset of the pandemic recreationists substituted their typical recreation destinations for areas more proximal to their homes such as neighborhood or city parks and away from more traditional destinations such as state, county, and regional parks. While longitudinal data collected for both measures showed trends back toward typical recreation patterns of distance traveled and type of area visited, there is evidence that the pandemic may have slightly shifted recreation visitation patterns toward local destinations.

Due to the unpredictable and changing nature of the pandemic, maintaining operations while rethinking strategies for recovery is difficult (Alexander et al., 2020; Groysberg & Abbott, 2020; Sibony, 2020). As a result, OAEE program managers and organizational leaders often make numerous decisions regarding program operations under stressful conditions and in response to guidelines from local, state, and federal governments as well as private and commercial industry.

## Methods

The purpose of this study was to ascertain the impacts of the COVID-19 pandemic on the OAEE fields and identify how OAEE organizations have responded to those impacts. A multi-phased study design was used to examine how the OAEE fields in the United States and elsewhere have been impacted by the COVID-19 pandemic during 2020. Using a multi-method design, employing both a survey instrument and interview process to collect quantitative and qualitative data, allowed for a complementary approach during data analysis as advocated by Creswell and Creswell (2018).

We asked four research questions to examine specific impacts from the COVID-19 pandemic on responding to OAEE programs. These impacts included variables that have influenced program viability, program manager concerns, and those factors related to COVID-19 that program leaders felt most impacted their program viability. The following research questions guided this project:

- RQ1: What are some of the ways in which OAEE programs have been impacted, for example, required to close, loss of staff, or loss of clients, by the emergence of COVID-19?
- RQ2: What specific variables, for example, organization size, age of the organization, or age of clients, have been influential in OAEE operations?
- RQ3: What are the OAEE management concerns related to COVID-19 as a function of program type?
- RQ4: What factors related to COVID-19 did OAEE program leaders perceive as having an impact on program viability during the pandemic?

### Sampling Framework

When the study was initiated, we intended that the representative responding to the study be an individual within the upper levels of the organization's leadership who could adequately assess and address the impact of the pandemic on their organization, including its future direction. This study utilized a multiphase framework and collected data using telephone interviews, a survey instrument, and document examination. The multiphase approach allowed researchers to gather data over time and to make adjustments to the instrument as needed between phases, and within each phase, each person in our sample had the opportunity to respond.

### Data Collection

Data collection was divided into four sequential phases, with Phases 1–3 designed to capture information from the beginning of the pandemic through 2020. Phase 4 is designed to continue data collection into 2021 and is not included in this analysis. The research was approved by the Prescott College Institutional Review Board. All survey instruments are available upon request to the lead author.

#### Phase 1

Phase 1, completed by May 6, 2020, provided a grounding for understanding how organizations were dealing with the pandemic and to inform the design of Phases 2–3 of the survey instrument. Phone interviews were conducted with leadership staff from 12 OAEE organizations to gather general information about the state of their programs during the initial months of the coronavirus pandemic. The 12 organizations in the Phase 1 sample were selected using a convenience sample (Creswell & Creswell, 2018). From these interviews four themes of organizational concern emerged: (a) safety and risk management; (b) operations, logistics, and financial solvency; (c) the nature of outdoor programming; and (d) changing clientele and staff. These themes informed the data collection in Phases 2–3.

#### Phase 2

**Figure 1.**
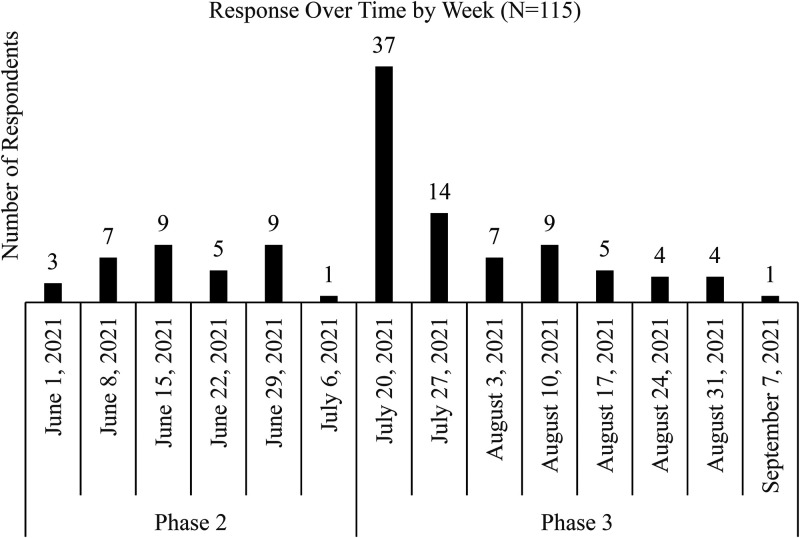
Phase 2 and 3 survey response timeline.

Phase 2 data collection began in June 2020 and ended in July 2020 (Figure 1). Surveys were emailed to 65 organizations accredited by the Association of Experiential Education and 18 organizations from the Association of Outdoor Recreation and Education identified as past recipients of the David J. Webb Program Excellence Award. The sample size was 73 with 34 usable responses and represented a wide range of OAEE program sizes and types. Respondents were grouped into three organization types: (a) outdoor and adventure education programs (OAE); (b) outdoor behavioral healthcare (OBH), whose primary purpose is the prescriptive use of wilderness experiences by licensed mental health professionals to meet the therapeutic needs of clients (OBH Council, n.d.); and (c) schools, colleges, and universities (SCU). Verification of types was completed through document analysis of organization websites, program mission, operational statements, and program outcomes.

#### Phase 3

Phase 3 data collection began in July 2020 and continued through September 2020 (Figure 1) and resulted in 81 useable responses. The Phase 3 survey instrument was fielded to members of the Association for Experiential Education (AEE) and the Association of Outdoor Recreation and Education (AORE), and past participants of the Nature's Grace Symposium, held at the University of Utah in 2020, as well as posted to social media accounts for AEE, AORE, and other organizations and individuals. A slightly modified version of the Phase 2 survey was used in Phase 3 with two questions edited for clarity and a question concerning systemic injustice and inequality issues made more visible by the pandemic added.

### Data Analysis

Phase 2 data and Phase 3 data were analyzed both in aggregate and as separate sets (N = 115; 34 from Phase 2 and 81 from Phase 3). Quantitative analysis included descriptive statistics and frequencies with qualitative data being codified using an exploratory method of holistic coding during the first cycle (Dey, 1993; as cited in Saldana, 2013, p. 142). Coding, in all cycles, was conducted using computer-assisted qualitative data analysis software (CAQDAS). Holistic coding allowed researchers to prepare for second cycle coding by getting a sense of direction from the data before assigning more specific codes. Holistic coding was conducted by the primary coder and was reviewed by several other researchers “to incorporate a range of alternative tools that can facilitate making sense of different kinds of data” (Colley & Diment, 2001, p. 14).

An eclectic method of evaluation and emotion coding was developed for second cycle coding (Goleman, 1995; as cited in Saldana, 2013 p.105; Miles et al., 2020; Prus, 1996). Evaluation coding helped researchers interpret data considering specific evaluative criteria. For example, if an organization was financially resilient throughout the pandemic, the data indicating financial resiliency was coded “+ Financial: Successful.” In addition, evaluation coding was a useful strategy in understanding respondents’ programs and policies both prior to and during the COVID-19 pandemic. Emotion coding was used to identify data that addressed RQ3. For example, researchers used emotion coding to gain a better understanding of how respondents perceived national leadership concerns during the pandemic. Respondents said things like, “The leadership of our country is not taking the threat seriously,” which was coded, “fear – not taking COVID-19 seriously.” The use of an eclectic method of coding allowed researchers to use different lenses to analyze data and allow more nuanced themes to arise in the second cycle of data analysis. Second cycle coding was conducted by three authors separately, major themes were cross-checked and confirmed. While some disparity existed in codes that had a lower frequency, all major themes were similar when cross-checked between coders.

Member checking as a form of verification, though widely used (Creswell & Creswell, 2018; Creswell & Poth, 2018), has weaknesses (Morse, 1998) that apply to this project. Given that our project has a large sample and covers several subdisciplines of OAEE, it was difficult for any single respondent of the study to provide verification outside of ascertaining the accuracy of a particular quote. Therefore, we asked four program managers to act as a theoretical member-checking group (Morse, 2018). The selected people have the expertise to accurately evaluate the findings of this study and provided a layer of verification that strengthens the validity of our claims.

## Findings

### Research Question 1: What are Some of the Ways in Which OAEE Programs Have Been Impacted by the Emergence of COVID-19?

#### Program type by operational Status (open or closed)

**Figure 2.**
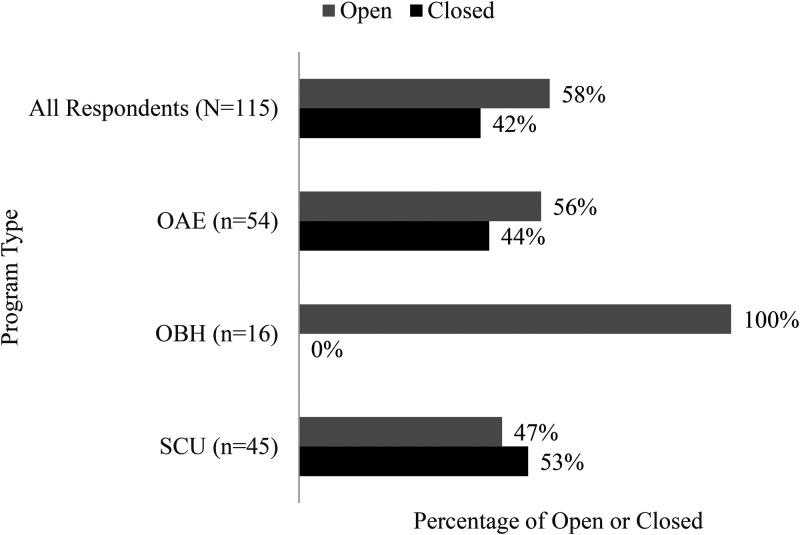
Program type by operational status attributed to the coronavirus disease-2019 (COVID-19) pandemic (open or closed) as of September 7, 2020.

*Note.* Operational status in Figure 2 indicates that a program reported being open or closed at the time they completed our survey. It does not indicate current program operational status.

The effects of COVID-19 caused many OAEE programs to close. As depicted in Figure 2 using Phase 2 and 3 data, 42% of respondents collectively reported being closed at the time of the survey. The data in Figure 2 (N = 115) indicate open or closed by program type: (a) 44% of outdoor adventure and education (OAE) program managers reported their programs were closed at the time they completed our survey (n = 54); (b) outdoor behavioral healthcare (OBH) program managers reported no closures (n = 16); and (c) schools, colleges, and universities (SCU) program managers reported 53% of programs were closed at the time they completed our survey (n = 45).

Data on closures represent a single point in time and do not indicate the length of closure—programs that reported closures may have been closed for a short time or a long time.

A total of 33% of SCU programs that reported being closed reported likely reopen dates while 18% were unsure of when they would reopen. Up to 37% of OAE programs that reported being closed reported likely reopen dates while 7% were unsure of when they would reopen.

#### OBH

All OBH programs (n = 16) reported that they were operational at the time they completed our survey. This may be influenced by the decision made by the CDC, state, and local governments to include mental health providers as essential services. This was the only program type surveyed that did not report closures. There was no indication that OBH programs were better prepared for a crisis or were more resilient organizations when compared to OAE and SCU programs. In some states, these programs were designated essential with OBH program managers indicating they responded to high market demand during 2020.

#### SCU

SCU program managers reported a higher percent of closures (53%) than OBH (0%) or OAE (44%) programs. Responses from program managers indicate this may be due to their often not having full administrative control over SCU programs as they are subject to state policies and guidelines as well as administrative decisions made by SCU leadership. A total of 33% of SCU program managers who indicated they were closed at the time they completed our survey also indicated they would attempt to reopen during the Fall 2020 semester, often planning to offer new and creative options such as on-campus outdoor activities and self-guided solo adventures into local parks and other public lands. Of these closed programs, 18% were unsure of when they would reopen.

#### Staffing level impacts attributed to the COVID-19 pandemic

**Figure 3.**
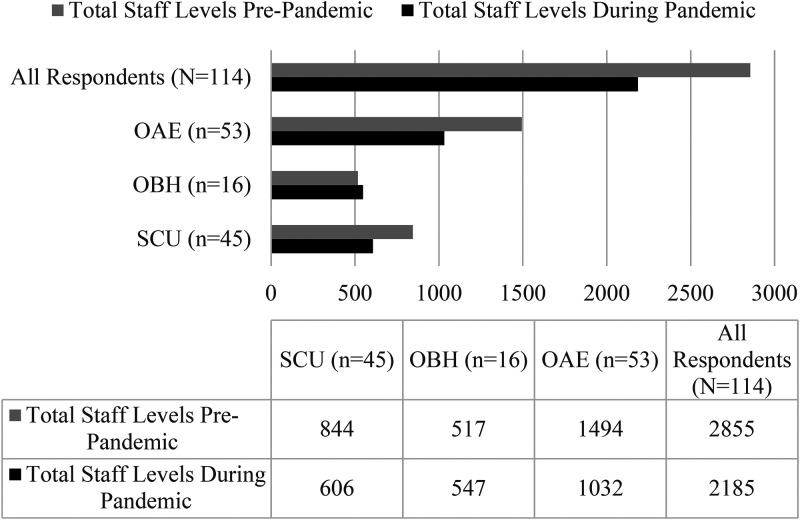
Total staffing level impacts attributed to the COVID-19 pandemic as of September 7, 2020.

Note. Total staffing levels reported at time of survey.

Another indicator that COVID-19 has impacted OAEE programs is change in staffing levels. As illustrated in Figure 3, overall staffing levels decreased by 23%. OBH programs reported a slight increase in staffing levels during the pandemic. OAE and SCU programs reported decreases in staffing levels of 31% and 29%, respectively, at the time they completed our survey compared to staffing levels prior to the pandemic. Respondents indicated that the loss of staff was worrisome as it both impacted the staff member and may hinder program reopening if staff are no longer available.

Respondents also indicated that their outdoor activities staff often require specialized training, and in some cases specialized skill certification. OAE and SCU programs, often with broad activity portfolios equating to needing a greater amount of outdoor activity skillsets from their staff, may experience challenges rehiring staff at a future date.

### Research Question 2: What Specific Variables Have Been Influential in OAEE Operations?

#### Program operational Status relative to population served

**Figure 4.**
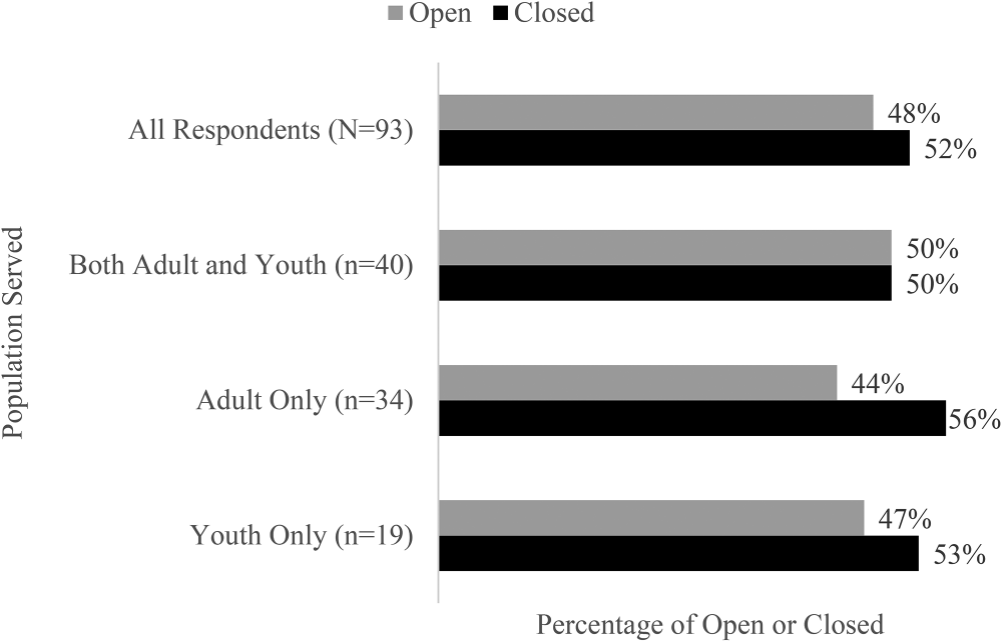
Outdoor adventure and experiential education (OAEE) program operational status relative to population served as of September 7, 2020.

Note. Operational status reported as open or closed at time of survey. Youth is classified as under 18 years, adult as 18 years and older.

RQ2 examined the influence of three variables that impacted OAEE operations: population served, year the organization was established, and number of people served. Figure 4 illustrates the effect of the pandemic on programs by populations served (adults, youth, both). There is no indication that populations served influenced the operational status of programs included in our study.

#### Program operational Status relative to the Age of the organization

**Figure 5.**
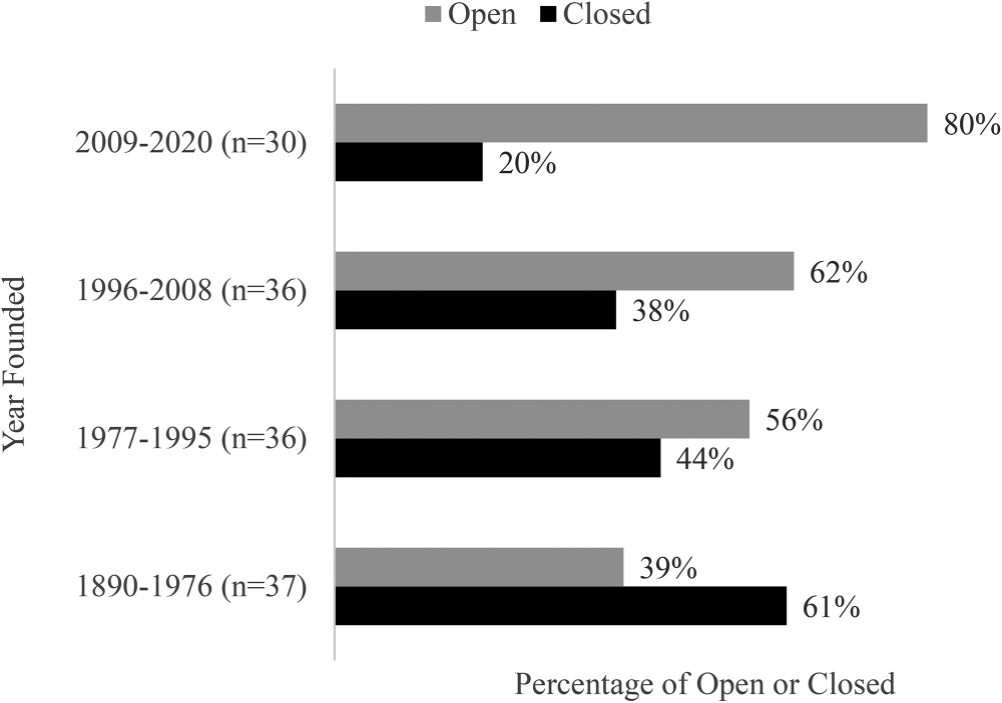
Program operational status relative to the age of the organization as of September 7, 2020.

Note. Operational status reported as open or closed at time of survey.

Data related to longevity (i.e., when was the organization first established), depicted in Figure 5, suggest that the older organizations were more likely to be closed at the time they responded to our survey. There was no indication that organizational longevity affected level of organizational preparedness (e.g., raised or lowered organizational resilience).

#### Operational Status relative to program size

**Figure 6.**
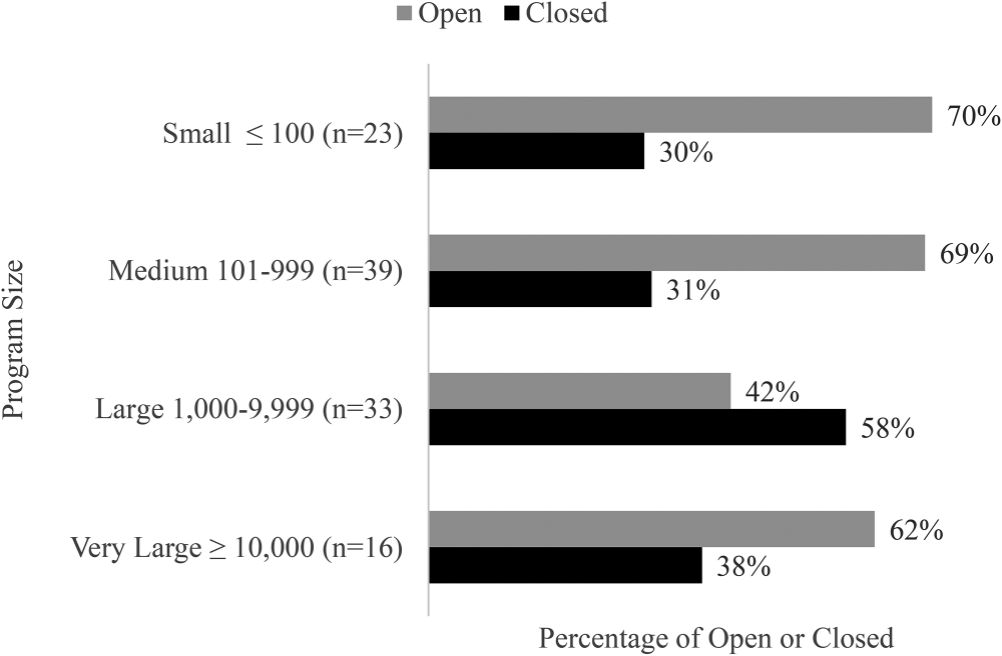
Operational status relative to program size measured as a function of client participants as of September 7, 2020.

Note. Operational status reported as open or closed at time of survey.

Figure 6 illustrates operational status relative to program size measured as a function of the number of client participants. Data suggest a bimodal distribution with organizations having client populations between 1,000 and 9,999, reporting higher rates of closure than those organizations with client populations over 10,000 and for those with fewer than 1,000 clients.

### Research Question 3: What are the OAEE Management Concerns Related to COVID-19 as a Function of Program Type?

Program managers responded with many concerns related to the COVID-19 pandemic with program viability, financial viability, and risk management at the top of their list across all three program types. The data suggest that the impacts to OAEE programs due to COVID-19 were variable dependent on the program type, for example, OAE, OBH, or SCU.

The impacts of COVID-19 are immense, and program managers’ growing frustration is clear. Apart from OBH programs (n = 16), which were deemed essential and reported no closures, 53% of SCU (n = 45) and 44% of OAE (n = 54) respondents reported being closed at some point between June and September 2020. One OAE program manager responded, “Well, it was a complete shutdown on a very short notice. As with so many places, we initially thought we would close the office for two weeks. Currently [July 2020] we have 1–4 people present on any given day and others working remotely. Work is a fraction of what we projected for 2020 so the financial implications are huge.”

#### SCU

Due to large institution leadership structures that often pass down mandates from top administration leadership, SCU program managers reported having little autonomy when deciding how to respond to the pandemic as well as little control over operations and finances beyond their immediate responsibilities. One university program manager stated, “Financially it has been a total headache and costly to untangle all the finances of canceling programs.” Another SCU program manager said, “I don’t think there was any way to greatly prepare for this pandemic programmatically in terms of how we usually operate. We did not have the resources or authority to continue providing programs.” SCU program managers reported a higher degree of concern for overall program viability. They were also concerned with the effects reducing operations or closing their program may have on students (their clients) and the overall health of the OAEE fields. At some point between May 2020 and September 2020, 53% of SCU programs were closed, and we suspect that the number of closed programs fluctuated (after we completed data collection for Phase 3 in September 2020) as universities determined if and/or how to reopen during the Fall semester of 2020.

#### OBH

Primarily at the early stages of the pandemic, there was a significant management concern regarding financial viability. However, OBH programs continued to operate throughout all three phases of data collection, and many increased their number of clients. Technology use grew to include telehealth and other virtual activities and virtual nature-based experiences, especially during the often-required 2-week quarantine phase when a new client entered the program. OBH program leaders reported being well connected with other OBH organizations and were consistently looking for ways to continue to support their clients through innovation. Program manager concerns were primarily focused on client care, staff retention, and their ability to meet program outcomes while mitigating the risk of transmission.

#### OAE

The most common management concerns in OAE programs between June and September 2020 were how to respond to the pandemic and remain open. In the sample, 44% of programs reported closing operations at some point between May 2020 and September 2020. OAE programs reported the need for risk management solutions, program or outdoor activity options, transportation accommodations, and access to resources that would help them remain viable until, as one respondent stated, “things get back to normal.” OAE program managers often reported the use of message boards from professional membership organizations and associations such as the American Camping Association, the AEE, and the Association for Outdoor Recreation and Education, to help them solve emergent problems.

When asked to share what they were doing to reopen, one OAE program manager simply responded “What can I pivot to? More day programming?” Managers indicated that preventing disease transmission was the primary concern, which necessitated radical changes in human resources, outdoor activities, lodging, food management, and transportation. Financial viability was also a primary concern except in cases where organizations benefited from endowments or access to other stable sources of funding. Toward the end of our Phase 3 data collection in September 2020, managers became increasingly concerned with staff retention, the ability to rehire furloughed staff, and maintaining staff credentials and/or training.

Although differences were noted between program types, as described above, many of the themes that emerged from the data were consistent across types. The following findings supporting Research Question 4 are more general and emerged across two or more of the program types. Program types are mentioned when it is needed to situate data in the correct context.

## Research Question 4: What Factors Related to COVID-19 did OAEE Program Managers Perceive as Having an Impact on Program Viability During the Pandemic?

Program managers were asked about a variety of factors that may have impacted the program's ability to remain viable during and after the pandemic and their responses were organized into three themes: (a) risk management; (b) readiness; and (c) the changing nature of OAEE programming.

### Risk management

Risk management during the pandemic was a critical concern for leadership. Respondents indicated that the immediate need to reduce or eliminate potential virus transmission during the start of the pandemic created initial chaos. Because existing risk management plans were not designed with COVID-19 in mind, program managers initially relied on organizational leaders and state and federal government guidance and direction. Their early concerns included staff and client safety, community safety, how to respond to a client or staff member developing symptoms, and how to safely mitigate COVID-19 transmission. Operational impacts from risk management decisions included alterations to group transportation, sleeping, and eating procedures and a need to adapt outdoor activities to COVID-19 standards. In some cases, organizations with larger outdoor activity portfolios reported having greater success in adapting to COVID-19 standards because they had multiple activities that could be more easily modified.

### Readiness

Out of 77 responses to the question “Discuss ways in which you (your program) were prepared for this pandemic?” A total of 48% suggested that they or their organization were not prepared or could not identify a way in which they were prepared, 3% of responses were neutral, and 49% were able to provide some way in which they felt that they or their organization were prepared. There was no significant difference between Phase 2 and Phase 3 responses.

Readiness in our study is an examination of an OAEE program's level of preparedness, an indicator of an organization actively building and maintaining resilience. Data from this study identified three elements of organizational resilience we used to determine levels of readiness: (a) financial reserves; (b) organizational leadership; and (c) standards and policies (Atella, 1999).

Programs that indicated some degree of readiness suggested they had (a) financial reserves that allowed for continued operations (even if at a reduced capacity), (b) operational leadership that was able to navigate the complexity of the pandemic, and (c) existing policies that either mitigated the impact of COVID-19 or were easy to adapt. For example, several programs already had work-at-home policies and respondents suggested that those policies made transitions easier. One organization stated that the procedures they developed during a prior pandemic enabled them to cope with the impact of the COVID-19 pandemic.

Programs that showed some degree of readiness also made decisions as needed including reducing staff, reducing capacity, redesigning and/or eliminating certain activities (e.g., adapting low ropes but eliminating high ropes), and immediately incorporating COVID-19 safety and risk management protocols as they were developed internally and adopted from other programs.

### The changing nature of outdoor programs

After concerns for the safety of participants and their communities, a common concern among program managers was losing the essence of outdoor adventure and experiential education. Many respondents felt that effective OAEE programs require face-to-face interactions that are relational and are not easy to translate to the virtual environment. One respondent said, “We are losing our most powerful tool of in-person experiential activities if we opt for virtual [programming] as the new norm.”

While the most common form of planned adaptation involved using different forms of virtual integration, several other themes emerged from the data about how organizations planned to adapt. Several of the respondents suggested that they would move their programs from distant destinations to local parks, nature areas, or urban areas. One organization suggested that they would focus on “sense of place, creating more opportunities to engage in local history.” Another respondent suggested that they would keep their program more local and would increase their focus on indigenous history of the area. One respondent suggested that the focus of their programming would be meeting students in their local places. This organization said that they plan on “shifting completely to short local trips offered throughout the country near our student's hometowns. We are no longer thinking about bringing students to us but utilizing resources in their own communities to organize programming for them.”

## Discussion

There can be little doubt that the COVID-19 pandemic has impacted the OAEE fields. Program closures, a significant decrease in enrollment, and staff reductions all followed the emergence of the pandemic. OAEE organizations confronting the pandemic found themselves faced with the realities of choosing whether to fire/furlough/retain employees and planning for reopening or permanently closing amid uncertainty.

Clearly OBH programs, identified as essential, were able to remain open while OAE programs responded to governmental orders and SCU programs responded to governmental orders and their higher administration. While there is data to support organizational age (younger organizations more likely to remain open) and size (bimodal distribution with smaller and larger organizations more likely to remain open) affected open and closure rates, of interest is how organizations responded to risk and what effect organizational readiness has had.

A total of 49% of organizations indicated some readiness for the pandemic, but none responded that they were fully prepared. Many organizations aggressively sought out information on how they should respond regarding risk management, looking to both professional organizations and other leaders in the field for solutions. This response may be due to OAEE program managers’ experience while understanding their simultaneous responsibilities to their staff, their clients, and to the health of their business.

OBH programs remained operational during 2020 and worked to meet their client's needs while coping with the organizational stress caused by the pandemic. While the OBH program managers who responded to our study indicated they had the same general concerns around program viability as the OAE and SCU program managers, they also responded with more general optimism for the future as they found ways to continue to provide services to their clients.

These challenges are magnified by what Worley and Jules (2020) characterized as a VUCA (volatility, uncertainty, complexity, and ambiguity) environment; that is, environments that are volatile, uncertain, complex, and ambiguous (U.S. Army War College, 2019). Or what Taleb (2007) referred to as a black swan event, those events that are “outside of the realm of regular expectations” and have the potential to “carry an extreme impact.” Facing this type of setting, the data in this study indicate that many OAEE organizations were ill prepared for the full breadth of operational, financial, and personal stress (staff and participants) caused by the pandemic. However, data suggest that if organizations had invested in pre-pandemic preparedness, they may have developed both a greater level of resistance to the conditions caused by the pandemic as well as a greater level of confidence that they would recover (Darkow, 2018). While black swan events are sometimes labeled unpredictable, this pandemic was predictable in the sense that experts knew there would be one sometime (Murphy et al., 2020). Additionally, Worley and Jules (2020) remarked that organizations’ preplanning helped them be agile and sustainable in the face of potentially volatile, uncertain, complex, and ambiguous environments.

## Implications

The findings from this study suggest organizations within the OAEE fields lacked a certain type of readiness to address and respond to a global pandemic strategically, financially, and organizationally. The impact of the readiness and response varied depending on the scope of the program.

Implications suggest that future research focus on organizational resilience, resistance to stress (e.g., resistance to stresses and management concerns caused by COVID-19) and organizational recovery during the post-vaccine rollout period. Resilience in this study is the result of pre-pandemic preparedness resulting in an organizations’ ability resist the stress (Ewert & Tessneer, 2019), whereas recovery occurs when an organization experiences adversity and begins to recover to a new level of operations when compared to pre-pandemic operations. Recovery may also result in achieving growth by attaining a higher level of operations and a greater sense of purpose (Davoudi et al., 2013) when compared to pre-pandemic operations. The findings from this research can be utilized to assess next steps and address issues related to organizational readiness, personnel recovery and growth, and governmental involvement.

This study also highlighted the vulnerability of personnel who design and deliver OAEE programs. These individuals (staff, facilitators, and trip leaders) are foundational for the OAEE fields; without them, programs cease to exist. Many OAEE organizations had to furlough or lay off staff, which challenges these individuals’ resiliency and ability to recover personally and professionally. Future research should look at what type of emotional, mental, spiritual, and financial support organizations provided to their staff during the pandemic, as well as what changes were implemented to hiring and retraining as programs began to offer services once again.

As OAEE organizations recover from the pandemic, the research from McKinsey & Company (Alexander et al., 2020) suggested that organizations need to create systems to foresee new threats, adjust existing practices, and become less static and more agile in their approach to risk management. This practice should touch all facets of an organization with examination and review of mission statement, administrative policies, staff training standards, and financial management among other aspects. Future research should aim to evaluate the long-term recovery changes OAEE organizations make in response to the pandemic, and if those changes had direct correlations to levels of resiliency, recovery, and growth.

Lastly, the study asserts that many OAEE organizations faced the reality of how a pandemic can devastate an operation when it relies primarily on providing in-person experiences. Examining research comparing how private companies and educational programs fared compared to local, state, or federal government supported OAEE entities, (e.g., policies, practices, funding) could provide guidance to how to prepare for future disruptions. This research could also aid OAEE organizations that suffered significant setbacks with relevant data which can inform new accepted practices, helping them transition from a recovery mindset to a growth mindset.

Questions remain as to what OAEE programs will look like in the future. Will programs remain essentially unchanged from before the pandemic? Or will they enact changes such as in program delivery, business models, and staff training protocols in response to COVID-19? Will organizations that recover from the impacts of COVID-19 pandemic operate at greater or lesser capacity? Will program outcomes change as a result of reaction to the pandemic (Ewert & Davidson, 2021). Moreover, in what ways can OAEE programs provide support and assistance in helping individuals and populations cope with the impacts of COVID-19?

## Limitations and Future Research

### Small Sample Size

There are a substantial number of OAEE organizations in the United States and tens of thousands more globally. Given the relatively small sample size, the data may not comprehensively reflect the broader population of OAEE organizations and practitioners.

### Data Collection Timing

This study utilized data collected between June 2020 and September 2020 (Figure 1). The pandemic's impact on the OAEE fields likely shifted during that time and will shift more as transmission mitigation and vaccines are released. The study reflects data from organizations impacted by the pandemic during the most uncertain and volatile time and not following the cessation or lessening of the pandemic. Phase 4 is designed to ascertain organizational coping and operations after the initial COVID-19 pandemic and may be substantially different from the first three phases.

In addition, the survey process did not include questions that could provide a comparison over time. That is, did organizations improve, stay the same, or diminish in their ability to deal with the pandemic. To account for the timing limitation, future research should involve tools such as a retrospective pre-test instrument (Faircloth & Bobilya, 2013; Moore & Tananis, 2009) measuring how various organizations believed they have changed or not in response to the pandemic.

It should also be noted that the respondents in all three phases were predominately from the United States (10 of 115 respondents were from outside of the United States). As such, these data may not be fully representative of a more international perspective and experience.

As noted in the research responses, preparedness and the ability to respond to a pandemic was not typically part of an organization's policy or procedures. The lack of precedent on how to adapt and manage an organization's strategic mission with new operational objectives during a pandemic left organizations without a model providing guidance on accepted practices and standards in response to risk mitigation, health, and safety issues, or how to manage fiscally during a long-term crisis. The lack of precedent coupled with an evolving long-term crisis, with critical national health and safety guidelines constantly changing, creates an environment where organizational responses and procedures can be obsolete within a few days.

Moreover, the research relied on an organization's representative to respond appropriately to the research questions. The data generated in the study were strongly based on anecdotal information and due to the accelerated nature of timing of the study, it is challenging to corroborate all the responses from the research participants to fully address the impact, implications, and corroboration of qualitative responses.

Due to the nature and the duration of COVID-19, especially as it is on the cusp of disrupting a second year of business priorities, future studies should include organizational resiliency, decision-making modalities, stringent qualitative analysis to include verification and affirmation of sources through peer review, and larger sample sizes of organizations within the outdoor industry.

## Conclusions

Participant responses present a compelling picture that the COVID-19 pandemic had a significant impact on many OAEE program's ability to operate as well as potentially on future operations. COVID-19 has exerted a broad spectrum of negative and often debilitating impacts across the globe. Organizations in the OAEE fields have been particularly vulnerable to a number of these impacts because they usually have a heavy reliance on face-to-face activities with program participants and are only partially amenable to online or other electronic meeting venues. Moreover, they can be influenced by several externalities beyond their control such as public health decrees and governmental-imposed lockdowns.

What is also clear is that many OAEE organizations in this study were unprepared for this crisis. It remains to be seen if the COVID-19 crisis recovery will substantially differ from other crises that the OAEE fields has experienced such as the Great Recession beginning in 2008 when enrollments significantly dropped. Or is this a VUCA environment (Worley & Jules, 2020) or a black swan event (Taleb, 2007) in which knowing what to do is exceedingly problematic, in part, because each crisis is different in important ways from each other.

Several important issues have emerged from this current crisis and that have ramifications to the OAEE fields. First is the structural inequity (e.g., financial, social, racial, and other issues of diversity) of the OAEE fields. Second, is whether and what the OAEE fields will learn from the COVID-19 experience. Once past, will we go back to business as usual or will programs and organizations change to be better prepared and able to respond in a nimbler manner? Learning these and other findings represents the difference between acting nimble out of necessity and acting nimble out of design. COVID-19 has caused some OAEE organizations to close, and it is hoped that the outdoor adventure and experiential educations fields will be better positioned to react to the next set of challenges that remain in the future.

This study demonstrates that OAEE programs can successfully operate in a COVID-19 environment and supports Quay et al. (2020) who provided a broad array of ideas and suggestions on how OAEE programs can adjust to a COVID-type setting. Those authors provided an international perspective on a variety of salient topics such as teaching effectiveness, organizational response, multiple stakeholders, and the need for future research.
